# Health Providers’ Perceptions and Experiences of Using mHealth for Chronic Noncommunicable Diseases: Qualitative Systematic Review and Meta-Synthesis

**DOI:** 10.2196/45437

**Published:** 2023-09-12

**Authors:** Yu Gu, Yushan Guan, Zhaolin Meng

**Affiliations:** 1 Yanjing Medical College Capital Medical University Beijing China; 2 School of Nursing Capital Medical University Beijing China

**Keywords:** mHealth, mobile health, health providers, adoption, chronic noncommunicable diseases, systematic review, meta-synthesis, mobile phone

## Abstract

**Background:**

Mobile health (mHealth) technology has great potential for addressing the epidemic of chronic noncommunicable diseases (CNCDs) by assisting health providers (HPs) with managing these diseases. However, there is currently limited evidence regarding the acceptance of mHealth among HPs, which is a key prerequisite for harnessing this potential.

**Objective:**

This review aimed to investigate the perceptions and experiences of HPs regarding the barriers to and facilitators of mHealth use for CNCDs.

**Methods:**

A systematic search was conducted in MEDLINE (via Ovid), Embase, Web of Science, Google Scholar, and Cochrane Library (via Ovid) for studies that assessed the perceptions and experiences of HPs regarding the barriers to and facilitators of mHealth use for CNCDs. Qualitative studies and mixed methods studies involving qualitative methods published in English were included. Data synthesis and interpretation were performed using a thematic synthesis approach.

**Results:**

A total of 18,242 studies were identified, of which 24 (0.13%) met the inclusion criteria. Overall, 6 themes related to facilitators were identified, namely empowering patient self-management, increasing efficiency, improving access to care, increasing the quality of care, improving satisfaction, and improving the usability of the internet and mobile software. Furthermore, 8 themes related to barriers were identified, namely limitation due to digital literacy, personal habits, or health problems; concern about additional burden; uncertainty around the value of mHealth technology; fear of medicolegal risks; lack of comfortable design and experience; lack of resources and incentives; lack of policy guidance and regulation; and worrisome side effects resulting from the use of mHealth.

**Conclusions:**

This study contributes to the understanding of the beneficial factors of and obstacles to mHealth adoption by HPs for CNCDs. The findings of this study may provide significant insights for health care workers and policy makers who seek ways to improve the adoption of mHealth by HPs for CNCDs.

## Introduction

### Background

Chronic noncommunicable diseases (CNCDs) are the leading cause of death and disability worldwide and have been recognized as a major challenge for achieving the World Health Organization’s 2030 Agenda for Sustainable Development [1]. The management of CNCDs is a pressing global concern. Simultaneously, the COVID-19 pandemic has greatly affected the provision of care to patients with CNCDs [2]. It has become critically important to find alternative methods to assist people in need of managing their chronic illnesses.

Mobile health (mHealth) refers to a type of health service that applies any mobile device, such as mobile phones, smartphones, PDAs, and devices that work on wireless technology or Bluetooth-compatible devices [3,4]. Owing to the portability of, instantaneous access to, and possibility of direct communication via mHealth, it is increasingly being incorporated into health services, inspiring new models of remote health care delivery and cost-effective solutions for chronic diseases, whose long-term nature and need for continuous monitoring can be positively impacted [5]. Evidence regarding the use of mHealth interventions in improving adherence to treatment; maintaining appointments; collecting data; promoting lifestyle changes [6]; and supporting health providers (HPs) with remote patient care, real-time clinical reference, and digital education is growing [7]. Simultaneously, evidence regarding the efficacy, effectiveness, economics, and clinical preferences of mHealth in the treatment of many chronic diseases is growing [8]. Its performance in extending the reach and capacity of overburdened health care systems attracts immense attention from academics and industries worldwide [9].

Although increasing evidence has shown the potential facilitators of the use of mHealth for CNCDs, the introduction of mobile technology in a medical context is not without challenges [10]. The lack of reimbursement, outcome uncertainty, and data security breaches have undermined the use of mHealth for CNCDs [10-12]. There are still some concerns held by HPs, and many of them have mixed attitudes toward the adoption of mHealth [13,14]. As HPs are the gatekeepers of health services, understanding their perspectives is crucial for the digital transformation of the health care system and the improvement of health care delivery [14].

Several reviews have attempted to investigate the factors impacting HPs’ adoption of mHealth. However, to the best of the authors’ knowledge, no comprehensive qualitative meta-synthesis has been performed to understand HPs’ perceptions and experiences of the use of mHealth for CNCDs. Qualitative meta-synthesis based on the findings of multiple qualitative studies can identify both common and differential factors among studies and generalize their findings to better inform decision-making [15]. Two recent meta-synthesis reviews [16,17] aimed to understand people’s perceptions of mHealth use, but neither covered HPs’ perspective, with one study by Vo et al [16] from the patients’ perspective and the other by Eisapareh et al [17] from the users’ perspective.

### Objectives

This study aimed to conduct a qualitative meta-synthesis to identify the factors that HPs perceived or experienced as facilitators of or barriers to mHealth use for people with CNCDs.

## Methods

### Study Design

The systematic review and meta-synthesis were conducted in accordance with the updated version of the PRISMA (Preferred Reporting Items for Systematic Reviews and Meta-Analyses; 2020) checklist (Multimedia Appendix 1 [18]). The review protocol was registered with the International Prospective Register of Systematic Reviews (PROSPERO, CRD42022352872).

### Search Strategy

A systematic literature search was conducted in the MEDLINE (via Ovid), Embase, Web of Science, Google Scholar, and Cochrane Library (via Ovid) databases. The search terms included 3 categories of keywords: HPs, mHealth, and adoption. All databases were searched from their inception to July 23, 2022, and an updated search was performed on July 19, 2023. The full search strategy for each database is presented in Multimedia Appendix 2. In addition, the reference lists of the included studies and studies cited in previous reviews were screened to identify additional studies.

Studies were required to meet a set of inclusion criteria. Textbox 1 summarizes the inclusion and exclusion criteria.

**Inclusion and exclusion criteria.**

**Inclusion criteria**

- Participants: health providers (HPs; eg, clinicians, physicians, doctors, residents, nurses, general practitioners, and other health professionals)
- Phenomena of interest: the overall experiences and perceptions of the adoption or use of mobile health (mHealth) for chronic noncommunicable diseases among HPs
- Context: HPs at the hospital, home, or health care facilities
- Study: qualitative studies and the qualitative components of mixed methods studies published as full-text articles in peer-review journals
- Language: English

**Exclusion criteria**

- Focused only on patients, caregivers, or technology providers
- Quantitative studies, conference abstracts, case reports, protocols, and reviews

### Identification of Studies

All retrieved records were exported to EndNote (version X9; Clarivate). After excluding duplicates, titles and abstracts were independently screened by 2 reviewers according to the inclusion and exclusion criteria. All abstracts that could potentially meet the inclusion criteria were forwarded to full-text review. Any disagreements between the 2 reviewers over eligibility were reconciled through discussion with a third researcher. Reasons for exclusion were recorded for all excluded studies.

### Data Extraction

Data extraction pro forma was developed based on the Joanna Briggs Institute Qualitative Assessment and Review Instrument (JBI-QARI) data extraction tool [19], and the following data were extracted for each of the included studies: title, authors, date, geographical or cultural setting, participant characteristics, type of mHealth technology, data collection and analysis approaches, and relevant primary qualitative data (themes and quotations). Data extraction was performed by 1 reviewer and checked by a second reviewer.

### Quality Assessment

Studies that met the inclusion criteria were appraised for quality by 2 independent reviewers using the standardized critical appraisal instrument from the JBI-QARI [19]. The JBI-QARI comprises 10 questions, each of which is answered with no, yes, or unclear. The final scores were computed according to the number of *yes* items and rated as follows: low (0-4 items), moderate (5-7 items), and high (8-10 items) [20]. Studies rated as low were excluded. Any disagreement between the reviewers was resolved through a discussion with a third reviewer.

### Data Synthesis

This study applied a thematic approach to the qualitative meta-synthesis described by Thomas and Harden [21]. The process has 3 stages. First, the free codes were identified line by line according to the *results* or *findings* part of the primary studies. Second, these codes were grouped by comparing their similarities to create descriptive themes. Finally, the descriptive themes were repeatedly checked, and new conceptions, understandings, or assumptions were identified. In this stage, analytic findings (themes and subthemes) were generated, which presented the key findings of the primary studies and provided new views of this field. All 3 stages were completed by 2 reviewers. The data analysis process was subsequently checked to ensure the congruence of the interpretations and the adequacy of the analytic themes.

## Results

### Study Identified

**Figure 1:**
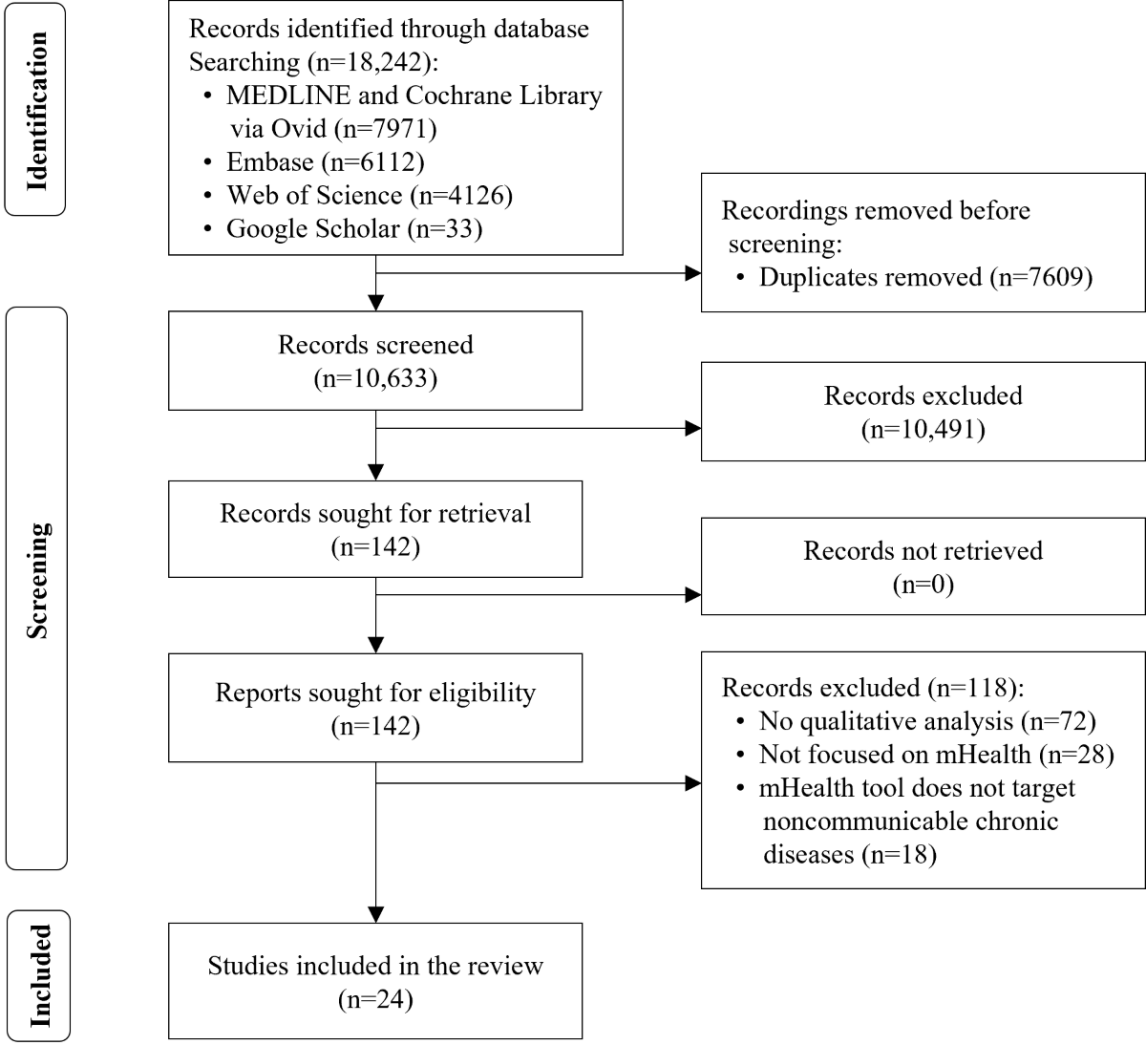
Flowchart of the included studies. mHealth: mobile health.

The database search and hand searches identified 18,242 articles, from which 7609 (41.71%) duplicates were removed. After screening the titles and abstracts, 142 (1.87%) full-text records were reviewed, of which 24 (16.9%) were included in the review. The study selection process is illustrated in Figure 1.

### Characteristics of the Included Studies

The characteristics of the 24 studies included in this review are described in Table 1. mHealth technologies were applied to assist the management of the following CNCDs: mental diseases (depression, anxiety, epilepsy, or multiple sclerosis; n=9, 38%) [22-30], cancer (n=2, 8%) [31,32], heart failure (n=4, 17%) [33-36], asthma (n=1, 4%) [37], diabetes (n=2, 8%) [38,39], chronic pain (n=1, 4%) [40], chronic obstructive pulmonary disease (n=1, 4%) [41], and other chronic diseases (n=4, 17%) [42-45]. The participant sample sizes ranged from 8 to 43. Studies were conducted in 13 countries, namely Ghana [38], the United Kingdom [22,29], Canada [33,36,41], Australia [28,31,34], Ireland [23], Nepal [24], Germany [25], the United States [26,32,35,37,39,40], France [27,42], the Netherlands [43], Sri Lanka [44], Spain [30], and China [45]. The studies were conducted in public or private hospitals, primary care institutions, and home care facilities in urban or rural settings. Data were collected in these studies through semistructured interviews (18/24, 75%) [23-26,28,29,31-36,38,41-45], focus group discussions (8/24, 33%) [22,24,27,28,30,37,40,42], or in-depth interviews (2/24, 8%) [37,39]. The analytic approaches followed in these qualitative studies included thematic analysis (11/24, 46%) [22-24,28,29,31,32,34,35,38,43], content analysis (7/24, 29%) [26,27,33,36,40,41,45], constant comparative method (4/24, 17%) [37,39,42,44], generic inductive approach (1/24, 4%) [30], and no specific method (1/24, 4%) [25].

**Table 1:** Characteristics of the included studies (N=24).

Author, year	Illness	Participants	Sample size	mHealth^a^ technology used	Data collection and analysis approaches	Setting and location
Korsah et al [38], 2023	Diabetes	Professional nurses	13	mHealth app (SMS text message)	Semistructured interview; thematic analysis	District hospital in Ghana
de Angel et al [22], 2022	Depression	Clinicians	6	Remote measurement technologies	Focus group; thematic analysis	Community in the United Kingdom
Sivakumar et al [33], 2022	Heart failure	Cardiologists, nurses, and nurse practitioners	21	mHealth apps	Semistructured interviews; content analysis	Heart failure clinic in Canada
Bezerra Giordan et al [34], 2022	Heart failure	Primary care clinician	6	mHealth apps	Semistructured interview; thematic analysis	General practice clinic in Australia
Melia et al [23], 2021	Mental diseases	Mental health professionals and clinician managers	15	mHealth	Semistructured interviews; thematic analysis	Primary care practices in Ireland
Pokhrel et al [24], 2021	Mental diseases	Primary health care workers and medical officers	43	mHealth	Semistructured interviews and focus group; thematic analysis	Primary care practices in Nepal
Dahlhausen et al [25], 2021	Depression	Physicians and psychotherapists	18	mHealth app (DiGA^b^)	Semistructured interviews; no specific method	Outpatient sector in Germany
Silfee et al [26], 2021	Anxiety or depression	Behavioral health and physical health providers	19	mHealth apps for cognitive behavioral therapy	Semistructured interviews; content analysis	Rural and urban primary care practices in the United States
Patoz et al [27], 2021	Depression	Psychiatrists and general practitioners	26	mHealth apps for depression	Focus groups; content analysis	Public and private sectors in France
Furness et al [31], 2021	Upper gastrointestinal cancer	Surgeons, nurses, oncologists, and dietitians	13	mHealth	Semistructured interviews; thematic analysis	Tertiary public and private hospitals in Australia
Sarradon-Eck et al [42], 2021	Multimorbidity	General practitioners	36	mHealth	Semistructured interviews and focus group; constant comparative method	Private practice in France
Portz et al [35], 2020	Heart failure	Physicians, nurses, social workers, and therapists	20	mHealth	Semistructured interview; thematic analysis	University hospital setting in the United States
Strodl et al [28], 2020	Mental diseases	Psychologist, nurse, general practitioners, and therapist	33	mHealth app (PTSD^c^ Coach Australia)	Semistructured interviews and focus group; thematic analysis	Public and Private sectors in Australia
Andrews et al [29], 2020	Epilepsy, depression, or multiple sclerosis	Doctors, nurses, clinical psychologists, physiotherapists, and dietitians	26	Remote measurement technologies	Semistructured interviews; thematic analysis	Health care organizations in the United Kingdom
Bally and Cesuroglu [43], 2020	Chronic disease	General practitioners, practice nurses, and insurers	18	mHealth	Semistructured interviews; thematic analysis	Primary care institutions in the Netherlands
Alwashmi et al [41], 2019	Chronic obstructive pulmonary disease	Nurses, pharmacists, and physicians	30	mHealth	Semistructured interviews; content analysis	Clinical setting (long-term care, community health, or clinic settings) in Canada
Han et al [44], 2019	Chronic disease	Health professionals	29	mHealth	Semistructured interviews; constant comparative method	Public or private hospitals in Sri Lanka
Anastasiadou et al [30], 2019	Mental health	ED^d^ specialists	8	mHealth app	Focus group; generic inductive approach	Public and private ED units in Spain
Berkowitz et al [32], 2017	Cancer	Physicians, advanced practice providers and supportive services providers	15	mHealth	Semistructured interviews; thematic analysis	Clinical practice in the United States
Chiang and Wang [45], 2016	Chronic disease	Community nurses	17	mHealth app (Line)	Semistructured interviews; content analysis	Home care facilities in China
Schneider et al [37], 2016	Asthma	Resident physicians and attending physicians	27	mHealth	In-depth interviews and focus group; constant comparative method	Academic medical center in the United States
Nundy et al [39], 2014	Diabetes	Primary care physicians and endocrinologists	12	Automated text messages by mobile phones	In-depth interview; constant comparative method	Primary care settings in the United States
Levine et al [40], 2014	Chronic noncancer pain	Primary care physicians and nurses	25	mHealth	Focus group; content analysis	Primary care settings in the United States
Seto et al [36], 2010	Heart failure	Cardiologists, nurse practitioners, and clinical fellows	16	Remote monitoring systems by mobile phone	Semistructured interviews; content analysis	Urban teaching hospital in Canada

^a^mHealth: mobile health.

^b^DiGA: *digitale gesundheitsanwendungen* (digital health applications).

^c^PTSD: posttraumatic stress disorder.

^d^ED: eating disorder.

### Assessment of Study Quality

The included papers were critically appraised and found to be of moderate to high methodological quality, with scores of 7 and 10 based on the 10 questions of the JBI-QARI (Multimedia Appendix 3 [22-45]). No study was excluded based on the quality critical appraisal tool. All studies showed congruity between the stated philosophical perspective and the research methodology (question 1). They were rated positively in terms of research methodology (questions 2, 3, 4, and 5). Among the 24 studies, the cultural or theoretical backgrounds of the researchers of 17 (71%) studies were inconsistently reported (question 6). Moreover, 17 (71%) papers rarely stated the influence of the researcher on the research (question 7), and 17 (71%) studies were granted formal ethics approval (question 9). All studies demonstrated adequate representation of participants’ voices (question 8) and concluded rationally (question 10). Overall, the methodological quality of the included studies was verified.

### Data Analysis and Meta-Synthesis

The results of the meta-synthesis are presented in this section. Data are presented as a synthesized finding with supporting themes and component subthemes. Summaries of themes and subthemes related to the perceived facilitators of and barriers to the adoption of mHealth by HPs are shown in Tables 2 and 3, respectively.

**Table 2:** Summary of the perceived facilitators from the reviewed articles with examples of quotations.

Themes and subthemes		Examples of quotations	References
**1.1. Empowering patient self-management**			
	1.1.1. Helping patients gain self-management knowledge	“Sometimes when we give advice, we don’t know what happens once they have gone home. If they have their application, it will support our advice about diet regimes, advice about care for certain chronic illnesses like diabetes...So, these are tools to help gain knowledge about their illness, to better understand the complications” [42].	[33,42]
	1.1.2. Making all-time remote monitoring possible and better	“With a self-administered survey on the app, the patient could do self-assessments. He could follow his clinical status and this could help him to realize ‘I feel better than last week’” [27].	[22,27,32,33,​^35^,^39^,^42^]
	1.1.3. Increasing adherence	“There’s a sense of accountability I believe from the patients. The nurse is watching me this morning, I better do it because she’ll be waiting or he’ll be waiting, definitely” [41].	[29,34,37-39,​^41^]
**1.2. Increasing efficiency**			
	1.2.1. Reducing workload and stress	“I prefer online interaction as it is quicker. I settle a lot online, which means I have less patients who visit me in person. While a GP^a^ consult normally is 10 minutes, in my practice I can spend 20 minutes on a face-to-face consult” [43].	[22,24,30,33,​^39^,^42^,^43^,^45^]
	1.2.2. Optimizing work procedures	“I think really it could have the potential to reduce the number of face to face contacts with children and increase the number of children who can access the service” [35].	[35,39,41,45]
	1.2.3. Facilitating clinical practice	“The app facilitates our clinical practice a lot...The whole team feels more reassured with regard to each patient’s treatment” [45].	[23,24,27,29,​^30^,^42^,^45^]
**1.3. Improving access to care**			
	1.3.1. Providing flexible care and promoting the continuity of care	“Clients could access support whenever they need it and wherever they are” [23].	[22,23,33,35,​^41^,^45^]
	1.3.2. Supporting family caregivers to participate in caring for patients	“Sometimes family members find that the patient has shortness of breath and feels strange, so they videotape it and send it to us...then we can ask the family members to follow our instructions to solve [the problem] step-by-step...we can show the caregiver how to do it, and we can also observe if the caregiver has done it correctly...Because of this, they feel more assured and do not feel the need to immediately visit the emergency room” [45].	[45]
**1.4. Increasing the quality of care**			
	1.4.1. Conveying accurate and credible information	“I [participant] do think apps could help a lot with...medication compliance, a lot of people that we care for are totally overwhelmed once they leave here, from all the information. Medication changes or dose changes, anything like that. So just something to keep them you know, on the right track once they leave here, especially if we are not going to see them for a few weeks” [32].	[24,26,30,32,33,​^36^,^39^,^42^,^45^]
	1.4.2. Allowing deeper and more timely data analysis	“If we can get data from SMS and other messaging apps, we will have, in the end, really precise clinical indicators” [27].	[27,30,34,35,​^38^,^42^]
**1.5. Improving satisfaction**			
	1.5.1. Promoting the physician-patient relationship	“Doctors and patients can communicate easily through mobile technology. It may be better than face-to-face communication. Sometimes patients feel uncomfortable to tell what they really think in front of doctors” [44].	[22,33,38,39,​^41^,^44^,^45^]
	1.5.2. Saving time and money	“For example, he told us that the patient had a surgical wound, very wet and exudated. He asked if the dressing could be removed...we then asked him to take a picture to show us. Because there was bleeding from the side, I told him that he needed to change the dressing, and also mentioned where the dressing could be purchased. Therefore, I did not need to go to their house and charge one additional visitation cost. [This way], we did not waste each other’s time, therefore saving time” [37].	[37,41]
**1.6.** **Improving the usability of the internet and mobile software**			
	1.6.1. Promoting the availability of reliable internet connections in the health facility	“Once I have a smartphone which most nurses have, what I need is the data and some tokens to be able to use it for our patients. Management can provide Wi- Fi in the wards so we could use it for nursing our patients” [38].	[24,38]
	1.6.2. Making the mHealth^b^ software easy to use	“It was very important for the app to be easy to use, such as by enabling automated self-monitoring through connected wireless devices instead of manual input of measures (eg, weight monitoring using a wireless scale connected to the app, physical activity monitoring using a fitness tracker)” [34].	[34,41]

^a^GP: general practitioner.

^b^mHealth: mobile health.

**Table 3:** Summary of the perceived barriers from the reviewed articles with examples of quotations.

Themes and subthemes			Examples of quotations		References
**2.1. Limitation due to digital literacy, personal habits, or health problems**					
	2.1.1. Limited digital literacy	“We certainly have a high number of elderly, or frail elderly in our clinic so a lot of them aren’t, you know, on email or internet or things like that” [33].		[24,26,28-31,​^33^,^40^,^41^,^43^]	
	2.1.2. Personal habits	“I think that probably technology maybe gets pushed to the side. I think that a lot of the physicians too might be, not scared but reluctant to use technology and to learn a new skill, especially if they’ve been in practice for thirty years or something” [41].		[31,40,41]	
	2.1.3. Health problems	“A lot of our patients may, especially if they’re more severely depressed, not be very motivated to interact with the app” [29].		[28,29,40]	
**2.2. Concern about additional burden**					
	2.2.1. Information overload	“When you get a 12 page report on one patient and you’re seeing 40 patients a day and you know time constraints with the amount of work that you do outside in terms of paperwork is already a burden” [41].		[25,29,40-42]	
	2.2.2. Excessive schedule pressure	“That the doctor pretty much has to do nothing [would make incorporating apps more feasible]. That’s the reality. I mean, the whole medical system, there’s more and more stuff you’re supposed to do and more and more paperwork and more and more time stuff, and so every new thing feels like a burden” [32].		[32]	
	2.2.3. Disturbing personal life	“One night you are snug in bed and you receive a text message ‘Mr. So-and-So has a systolic of 200!!’...you don’t sleep a wink all night!” [42]		[36,42,45]	
**2.3. Uncertainty around the value of mHealth** ^a^ **technology**					
	2.3.1. Lack of evidence of the value of mHealth intervention	“We [GPs^b^] won’t immediately implement the newest technologies. The technology should prove itself and earn our trust” [43].		[29,31,32,40,​^41^,^43^]	
	2.3.2. Dubious about the value of patient-gathered health data	“The validity of the data would be something that some people might question. I guess a lot of that would depend on how straightforward the devices are to use or how much training might be required to make sure that they are using it correctly” [41].		[29,41,42]	
	2.3.3. Concern about undermining traditional face-to-face services	“My [participants] fear would be that it would be used as a cost saving measure only and would undermine the quality of the service” [23].		[23,27,29]	
**2.4. Fear of medicolegal risk**					
	2.4.1. The potential for the misinterpretation of mHealth data	“The issue is [the] interpretation of messages received by the patient. You don’t have instantaneous feedback for it and if he had a wrong interpretation, it will not help him” [27].		[27,32,38,​^41^,^42^,^44^]	
	2.4.2. Legal liability for inability to immediately respond to an alert	“If we consider this as a medical practice, we need to be more cautious. If a practitioner tells patients that he [she] will provide them with Line [an app] communication and is unable to respond when patients send messages, the practitioner will be reprimanded. Offering this innovative service needs planning and a standard operating procedure, including details about who should handle it, and how long there is to respond” [45].		[27,29,36,45]	
	2.4.3. Inappropriate automated instruction	“The automatically generated instructions and alerts sent to the patients could be inappropriate. Some clinicians suggested that a clinician should vet each alert before the alert is sent to the patient” [36].		[36,42]	
	2.4.4. Data privacy and security	“How are patients confident that the information that’s in that app is only going to stay with them and that other people are not going to see that data?” [29]		[23,24,27-29,​^32^,^41^,^42^]	
**2.5. Lack of comfortable design and experience**					
	2.5.1. Non–user-friendly design	“I found the interface kind of clunky...it didn’t feel particularly user friendly...it was too wordy. There were lots and lots of words everywhere. The techniques that are in there are fine but I didn’t feel that it added value in terms of encouraging me to use it over other apps that I do use” [28].		[22,24,27,28,​^30^,^35^,^42^]	
	2.5.2. Lack of interoperability and integration	“And it’s not just having the training, it’s then having the time to think about that afterwards and incorporate it into your practice which would require a corresponding decrease in clinical word” [22].		[22,28,29,31,​^41^,^43^]	
	2.5.3. Technical glitches	“There’s been issues with the technology not communicating because we have setups in four different ways” [41].		[24,28,33,41]	
	2.5.4. Insufficient development support	“It is important to include the Dutch expert organization on eHealth as they set the standards for health information exchange. These specifications are necessary for developers of mHealth to build high quality solutions” [43].		[43]	
**2.6. Lack of resource and incentives**					
	2.6.1. Lack of financial investment	“I strongly think management needs to give us data or money for the data we would be using to enable us to use the application.” [38].		[31,38,40,41,​^43^,^44^]	
	2.6.2. Lack of workforce	“Outside of fixed appointments the question would be who would actually have time and headspace to actually look at what was being flagged up. You would need to really carefully think about the staffing in the NHS^c^ and mental health services” [29].		[29,33]	
	2.6.3. Lack of extra payment for health providers	“I mean we’re all so busy that nobody wants to do anything for free because why would I do that for free if I get paid for it” [41].		[33,37,41,45]	
	2.6.4. Inadequate medical insurance coverage	“Generally more patients with COPD^d^ are falling in the lower socio-economic grouping that wouldn’t necessarily be able to afford this” [41].		[22,41]	
**2.7. Lack of policy guidance and regulation**					
	2.7.1. Absence of policy on mHealth development	“Our employer doesn’t want to see us having them out, people will have the impression we are using it for personal use. That is one big factor. Our employer tells us, keep your phones hidden, don’t have them out” [41].		[28,30,43,45]	
	2.7.2. Absence of authorized certification for mHealth apps	“All available apps for health tracking should be authorised...they should be checked by health professionals because if such an app is not working well, it can bring even more damage” [22].		[22,30,42]	
	2.7.3. Absence of regulations on related legal responsibility	“I think that the dark side of this is the liability issue. If an adverse event does happen [and a provider does not respond appropriately], does it come back to bite us?” [40]		[27,35,40,45]	
	2.7.4. Absence of regulations on mHealth data protection	“Making sure mHealth data is not used for commercial purposes is something we [the government] can actively promote by setting the rules” [43].		[41,43]	
**2.8. Worrisome side effects resulting from the use of mHealth**					
	2.8.1. Worry that patients would become obsessed with their smartphone	“Some sub-groups of patients with anxiety might have impaired quality of life because then they become obsessed with that rather than actually just saying okay that’s what they’re saying, I’m okay” [41].		[22,23,41,42]	
	2.8.2. Worry that close contact between the patient and physician would be affected	“I like to have a bit of actual contact and eye contact, and hear the tone of someone’s voice, and a gentle touch sometimes can be so reassuring, you know. I think it’s going to be lost with this type of technology” [41].		[24,40-43]	
	2.8.3. Worry about the deepening of the social inequalities of health care	“Where I find it challenging, the people who need the resources the most are the ones who typically don’t have access to the resource...phones are getting cheaper, but still” [32].		[32,42,44]	

^a^mHealth: mobile health.

^b^GP: general practitioner.

^c^NHS: National Health Service.

^d^COPD: chronic obstructive pulmonary disease.

### Perceived Facilitators

The facilitators perceived by HPs were addressed in 22 (92%) of the 24 studies on the use of mHealth technology and included the themes of empowering patient self-management, increasing efficiency, improving access to care, increasing the quality of care, improving satisfaction, and improving the usability of the internet and mobile software (see Table 2).

#### Empowering Patient Self-Management

##### Helping Patients Gain Self-Management Knowledge

HPs felt that mHealth increased the accessibility of reliable self-management knowledge for patients to manage their chronic illnesses [33,42].

##### Making All-Time Remote Monitoring Possible and Better

HPs believed that mHealth provided them with a chance to assist patients with developing self-management skills to make symptom tracking and remote supervision more intensive and real time [22,27,32,33,35,42,43]. Furthermore, patient-collected information received via mobile phones may be more accurate than information collected during a clinic visit because monitoring at more frequent intervals can overcome the memory bias encountered in in-person follow-ups [27,39].

##### Increasing Adherence

HPs deemed that personalized feedback and automated messages contributed to improving patients’ motivation to manage their condition [34,38], and mHealth enhanced engagement by empowering patients to take a leading role in their care by increasing their sense of responsibility and self-efficacy to improve follow-up and therapy adherence [25,29,30,37,39,41,42].

#### Increasing Efficiency

##### Reducing Workload and Stress

Some HPs perceived mHealth as a tool that reduces workload and stress by reducing repetitive actions, expediting the transfer of information, and providing buffer time to properly consider how to handle and respond to a request [22,36,39,41-43]. In addition, HPs regarded mHealth as a useful reminder for HPs and patients on a day-to-day basis that provides convenient recordings to prevent forgetfulness [22,24,33,35,41,45].

##### Optimizing Work Procedures

HPs indicated that mHealth helped reduce unnecessary face-to-face visits and simplified in-person visits by allowing them to review medical recordings before seeing patients [35,39,41,45].

##### Facilitating Clinical Practice

HPs perceived that the reliability and objectivity of measurements and the traceability of measurement history of mHealth facilitated the provision of continuous and holistic care to patients [24,27,29,30,42,45]. In addition, HPs indicated that mHealth served as an instant, easily accessible resource for updating their skills and knowledge [23,24].

#### Improving Access to Care

##### Providing Flexible Care and Promoting the Continuity of Care

mHealth was perceived by HPs as helping patients obtain more flexible access to care independent of a practice’s opening hours and therapy location, especially in crises or for people in rural areas [23-25,33,35,41,45].

##### Supporting Family Caregivers to Participate in Caring for Patients

mHealth technologies were perceived by HPs as helping family caregivers deal with the chronic illnesses of patients by providing a method that facilitates timely and accurate communication of clinical information, which could avoid unnecessary medical visits [45].

#### Increasing the Quality of Care

##### Conveying Accurate and Credible Information

HPs felt that mHealth promoted accurate communication in chronic disease care, with a greater likelihood of improved outcomes [24,26,30,32,36,37,39,42,45]. For example, HPs perceived that mHealth provided patients with a means to share their symptoms and conditions via a photo and video transfer to prevent subjective judgment or differential perceptions, which might lead to communication errors [33,45].

##### Allowing Deeper and More Timely Data Analysis

HPs thought that mHealth could help them obtain more actual and direct information about the conditions of their patients via open-ended web-based chat, particularly from those who find direct social interaction to be challenging [39], and obtain patients’ data anytime and anywhere, which promote their insights into treatment and prognosis [23,24,27,30,34,35,38,45].

#### Improving Satisfaction

##### Promoting the Physician-Patient Relationship

HPs believed that mHealth could benefit the physician-patient relationship by improving effective communication between them and has the potential to improve patients’ and physicians’ satisfaction [22,33,38,39,41,43-45].

##### Saving Time and Money

HPs indicated that mHealth could save time and money by avoiding unnecessary hospital and home visitation services and thereby might improve patients’ and physicians’ satisfaction by reducing financial and geographic barriers [26,30,37,41].

#### Improving the Usability of the Internet and Mobile Software

##### Promoting the Availability of Reliable Internet Connections in the Health Facility

HPs believed that network infrastructure, such as Wi-Fi, can be a motivator for them to use mobile apps to manage their patients [24,38].

##### Making the mHealth Software Easy to Use

HPs deemed that mHealth software needs to be simple for them to use; for example, the language should be basic, the software should be visually appealing, and automated self-monitoring through connected wireless devices should be enabled instead of manual input of measures [34,41].

### Perceived Barriers

Despite HPs perceiving some facilitators of the use of mHealth technology, they also perceived some barriers to mHealth use. Barriers were discussed in all the 24 included studies and emanated from 8 themes (see Table 3).

#### Limitation Due to Digital Literacy, Personal Habits, or Health Problems

##### Limited Digital Literacy

HPs thought that physicians, particularly primary care physicians in rural areas and older physicians, had insufficient knowledge about and experience in using digital health technologies [24,25,28,30,31,37,41,43]. In addition, HPs also believed that the lack of technical literacy was a barrier for patients to manage their disease using mHealth, especially for older patients or those with a low level of education [26,28,29,31,33,40,41].

##### Personal Habits

HPs felt that many of them would be reluctant to change their habits in general practices, especially older HPs [40,41,43]. Moreover, they also thought that some older patients would prefer face-to-face consultation [31,43].

##### Health Problems

In addition, HPs considered that some patients with severe physical or mental health problems would have difficulty in using mHealth devices [28,29,40].

#### Concern About Additional Burden

##### Information Overload

HPs believed that patient-reported or automatically generated data by apps would result in information overload, which would create extra work [25,29,37,40-43].

##### Excessive Schedule Pressure

HPs stated that they did not have further capacity to take on duties that mHealth use would add to their busy schedules, such as excessive demand for immediate processing, interpretation, and responses [25,29,32,36].

##### Disturbing Personal Life

HPs also expressed feeling a little conflicted about receiving patients’ messages or managing alerts during off-work hours (during nights, weekends, and vacations) [36,42,45].

#### Uncertainty Around the Value of mHealth Technology

##### Lack of Evidence of the Value of mHealth Intervention

HPs thought that some of their peers were skeptical about mHealth technology and uncertain whether mHealth solutions could meet the expectations of saving time and maintaining high-quality care [25,27,31,32,37,43]. They lacked the time and resources to fully explore and assess the available apps [43]. Simultaneously, they expressed that evidence regarding the validity, reliability, utility, effectiveness, and risk-benefit of mHealth from research or other health care workers was insufficient [25,29,40,41,43]. Therefore, it was difficult for them to differentiate bad apps from good ones [29].

##### Dubious About the Value of Patient-Gathered Health Data

HPs believed that it was difficult to guarantee the accuracy of patient-gathered health data [24,29,43]. Therefore, they considered the value and usefulness of the vast amount of patient-reported data to be doubtful [35,41,42]. They were inclined to see patients in person to conduct the tests themselves.

##### Concern About Undermining Traditional Face-To-Face Services

Moreover, HPs stressed that they were concerned about mHealth technology undermining and even replacing traditional services [23,27]. They also feared the risk of transferring their role to the tools [23,29,31].

#### Fear of Medicolegal Risk

##### The Potential for the Misinterpretation of mHealth Data

HPs believed that the significant amount of data that patients recorded in mHealth tools might lead to missing critical information [42] and that web-based communication might cause difficulties in determining whether the patient obtained the information correctly [27,38,41,43,44]. All these conditions increase the risk of the misinterpretation of mHealth data and unintended outcomes [32,42-44].

##### Legal Liability for Inability to Immediately Respond to an Alert

HPs expressed that the opinions on the time taken to respond to patient data and the modalities and frequency of app use were numerous and strongly diverged among end users [27,29]. They might even hold different perceptions of the same incident [27,45]. All these conditions could lead to unintended medical liability [36].

##### Inappropriate Automated Instruction

HPs indicated that mHealth might send irrelevant or non–evidence-based information [42] and even inappropriate automated instructions via an incomplete alerting algorithm, which would mislead the patient [30,36].

##### Data Privacy and Security

HPs stressed that data privacy and security were regarded as important issues. They feared the risk of patient data being exploited and commodified [23-25,27-30,36,37,39,41,42,45].

#### Lack of Comfortable Design and Experience

##### Non–User-Friendly Design

HPs expressed that the end users were unsatisfied with the design of some mHealth tools for reasons such as a boring appearance, outdated content, heavy text, language deficits, the lack of preset options, the lack of an option to provide voice input, the lack of symptom trend charts, scattered information, and the lack of compatibility with different mobile operating systems [22,24,27,28,30,38,42]. They felt that the app design was less personalized and could not adapt to user characteristics and preferences [27,30,35].

##### Lack of Interoperability and Integration

HPs believed that a key barrier was the lack of interoperability between mHealth devices and existing health care information systems or among mHealth apps, and they were concerned that using mHealth would increase their workload [22,28,29,31,43,44].

##### Technical Glitches

HPs expressed that potential technical glitches, such as freezing, crashing, equipment malfunction, password issues, and unstable internet connection, would make the end user lose interest in using mHealth [24,28,33,35,41].

##### Insufficient Development Support

HPs thought that the lack of motivation among information system developers to optimize the systems limited the speed of information exchange between mHealth technology and other information systems [25,43].

#### Lack of Resource and Incentives

##### Lack of Financial Investment

HPs deemed that in some developing countries, there was not enough financial investment to cover the costs of data storage, infrastructure establishment, the assessment of the impact of mHealth tools, the maintenance of mHealth tools, and the replacement of outdated mHealth tools [25,31,38,40,41,43,44].

##### Lack of Workforce

HPs indicated that they lacked time, and there was insufficient workforce available to respond to every message from patients using mHealth apps [29,31-33,36,41,44].

##### Lack of Extra Payment for HPs

HPs expressed that they had limited readiness to work outside their routine responsibilities without overtime compensation [25,33,37,41,43,45].

##### Inadequate Medical Insurance Coverage

HPs believed that some patients with lower socioeconomic status could not afford mHealth services without adequate insurance coverage [22,25,41,43].

#### Lack of Policy Guidance and Regulation

##### Absence of Policy on mHealth Development

HPs expressed that health authorities had little interest in making users aware of the possibilities of mHealth and in making policies to support the implementation of mHealth [23,28,30,36,43,45]. In addition, hospitals typically did not request them to provide mHealth-related services. Therefore, mHealth service provision was based on personal willingness [45]. HPs lacked a sense of urgency about the provision of mHealth service without explicit managerial instruction and a policy on the adoption of the technology.

##### Absence of Authorized Certification for mHealth Apps

HPs indicated that there was no requirement to obtain validation from an authorized organization before putting mHealth apps into use; therefore, it was difficult to prevent false claims on the effectiveness of certain mHealth products or services [22,23,30,41,42].

##### Absence of Regulations on Related Legal Responsibility

HPs were concerned that a lack of regulations or specifications clarifying definitions, including those of immediacy, emergency, standard operating procedures, optimal care pathways, and data transmission conditions, would create disputes and even medicolegal implications [27,31,35,37,40,45]. Furthermore, there was a lack of overarching organizational systems for accountability management [31].

##### Absence of Regulations on mHealth Data Protection

HPs indicated that mHealth lacked standards and specifications about who could be allowed access to patients’ health information and in which situations the data could be used, which would threaten patients’ privacy and data security [27,41,43].

#### Worrisome Side Effects Resulting From the Use of mHealth

##### Worry That Patients Would Become Obsessed With Their Smartphone

Some HPs were concerned that a number of patients would become obsessed with mHealth, and the overuse of mHealth apps might cause anxiety, suspicious feelings, or a false sense of security, which would create or worsen some symptoms and thus increase drug prescriptions and health care expenses [22-24,27,29,41,42].

##### Worry That Close Contact Between the Patient and Physician Would Be Affected

Some HPs were concerned that mHealth would make them become technicians and affect the humanistic care of patients [24,40-43].

##### Worry About the Deepening of the Social Inequalities of Health Care

HPs worried that mHealth could be used only by certain patients, depending on their income level, level of digital literacy, and their linguistic ability [32,42,44].

## Discussion

### Principal Findings

This meta-synthesis extends our understanding of HPs’ perceptions and experiences of adopting mHealth for CNCDs and highlights specific facilitators of and barriers to mHealth use. The main perceived adoption facilitators were categorized into 6 themes: empowering patient self-management, increasing efficiency, improving access to care, increasing the quality of care, improving satisfaction, and improving the usability of the internet and mobile software. The perceived barriers could be categorized into 3 major groups: individual factors (limitation due to digital literacy, personal habits, or health problems; concern about additional burden; uncertainty around the value of mHealth technology; and fear of medicolegal risks), technological factors (lack of comfortable design and experience), and social and economic factors (lack of resources and incentives, lack of policy guidance and regulation, and worrisome side effects resulting from the use of mHealth).

A previous review [46] suggested that 2 technical acceptance model factors [47], usefulness and ease of use of the technology, were seen as 2 of the most important factors with respect to the adoption of mHealth. In addition to the facilitator of the usability of mobile technology, our findings also showed that empowering patient self-management was a main perceived adoption facilitator for mHealth. The previous review [46] examined the factors influencing health care professionals’ adoption of mHealth tools that were not specifically designed for chronic diseases; however, this study focused specifically on mHealth tools for chronic disease management. In most of the included studies (16/24, 67%), HPs expressed that they were more likely to adopt mHealth when it could improve self-management in chronic care [42,47,48]. Moreover, mHealth could potentially improve patient health and minimize the need for office visits for the routine management of some of the most common acute and chronic issues because of greater patient self-management [49]. In addition, mHealth can increase the accessibility of care and enhance the monitoring, tracking, and communication of various biometric information [50]. HPs also indicated that mHealth could eliminate the time demands on physicians, which was associated with greater physician satisfaction.

Despite HPs perceiving some facilitators to the use of mHealth technology, they also perceived some barriers to mHealth use. In addition to the barriers to mHealth use at the individual and technological levels, which had been emphasized in previous studies [46,51], this study also highlighted the social and economic factors that were perceived by HPs as important barriers to mHealth use.

Privacy and security issues had been regarded as significant barriers to HPs’ adoption of mHealth. mHealth devices generate substantive amounts of personal data. HPs feared the risk of patient data being exploited and commodified [23-25]. Although many countries are developing legislations or regulations to deal with this concern [52], few mHealth apps received a comprehensive risk analysis before the trial to ensure data privacy and security [53]. For HPs, the assessment of individual apps and literature searches on app evidence are highly time consuming and challenging to perform on their own [54]. Given the importance of secure and private channels of communication, it was also found that some clinicians may be challenged in finding mHealth technology partners willing to sign business associate agreements for security and privacy [54]. These findings suggest that a more nuanced approach to privacy and security may be needed to support mHealth expansion.

Moreover, the reimbursement of tasks related to mHealth was also perceived as a potential barrier to mHealth adoption. This finding was consistent with a prior analysis of factors related to mHealth use [46]. Without payment, it would be difficult for HPs to afford to provide services to patients with chronic illnesses using mHealth [53]. However, it has been reported that many countries have not yet incorporated reimbursement approval [52]. These findings suggest that adequate reimbursement for mHealth may be a critical incentive for maintaining the broad adoption of mHealth.

Notably, HPs felt that mHealth could reduce their workload and stress by reducing repetitive actions. HPs also believed that patient-reported data collected using mHealth would result in information overload, which would create extra work [29,41,43]. HPs were also concerned about how managing the alerts could disturb their personal lives [36,42,45]. These findings suggest that specific regulations for the use of mHealth should be implemented and indicate the need for increased incentives for mHealth use.

Although HPs perceived that mHealth offers a way to address the barriers to care, they were also concerned about unequal access to mHealth for some patients due to low income and the lack of digital literacy, which may deepen the social inequalities of health care [7,55]. This potential side effect of mHealth has been less frequently reported in previous studies. Previous studies have also shown little evidence of the widespread use of mHealth in resource-poor settings [55,56]. Therefore, improving the implementation of mHealth in low- and middle-income countries is critical to meet unmet health care needs, especially among susceptible people, such as older patients and patients with poor digital literacy skills.

### Strengths and Limitations

To our knowledge, this is the first meta-synthesis to examine HPs’ perceptions and experiences regarding the barriers to and facilitators of mHealth use for CNCDs. The review was based on an extensive literature search and adhered to best practice processes to ensure rigor and quality, and bias was minimized in terms of the literature search, appraisal, and synthesis. Overall, the methodological quality of the included studies was verified based on the JBI-QARI critical appraisal tool.

This study has several limitations. First, all the studies included in this review were published in English, thus eliminating any important papers in other languages. Second, most of the included studies were from high-income countries. Therefore, the findings may not be representative of countries with different cultures and income levels. Third, the results of this study were based on a synthesis of qualitative studies, which is inherently subjective; however, the involvement of a review team improved the robustness of the findings.

### Conclusions

This synthesis provides an overview of the qualitative literature on HPs’ experiences and perceptions regarding the adoption of mHealth for CNCDs. The facilitators of the use of mHealth technology as perceived by HPs fell under the themes of empowering patient self-management, increasing efficiency, improving access to care, increasing the quality of care, improving satisfaction, and improving the usability of the internet and mobile software. The perceived barriers included individual, technological, social, and economic factors. On the basis of these findings, interventions are needed to address the identified obstacles to foster HPs’ adoption of mHealth for CNCDs. The findings of this study may provide significant insights for health care workers and policy makers who seek ways to improve HPs’ adoption of mHealth.

## Appendix

Multimedia Appendix 1 PRISMA (Preferred Reporting Items for Systematic Reviews and Meta-Analyses) 2020 checklist.

Multimedia Appendix 2 Search strategies.

Multimedia Appendix 3 Quality assessment of the included studies.

## Abbreviations

CNCD: chronic noncommunicable disease
HP: health provider
JBI-QARI: Joanna Briggs Institute Qualitative Assessment and Review Instrument
mHealth: mobile health
PRISMA: Preferred Reporting Items for Systematic Reviews and Meta-Analyses
