# Supplementary material for: Health Providers’ Perceptions and Experiences of Using mHealth for Chronic Noncommunicable Diseases: Qualitative Systematic Review and Meta-Synthesis
Source: J Med Internet Res. 2023 Sep 12;25:e45437. doi: 10.2196/45437 (PMC10523226; doi:10.2196/45437)
Supplement: Multimedia Appendix 2 [file jmir_v25i1e45437_app2.docx]

Multimedia Appendix 2. Search strategies

(Medline, Cochrane Central Register of Controlled Trials) Via Ovid

| **Search** | **Query** | **Results** |
| --- | --- | --- |
| 1 | exp Health Personnel/ | 626430 |
| 2 | (Anesthetists or Dentists or "Medical Staff" or Nutritionists or "Occupational Therapists" or Pharmacists or "Physical Therapists" or "Allied Health Personnel" or Anatomists).mp. | 168604 |
| 3 | ("personnel, health" or "health care provider*" or "provider, health care" or "provider, healthcare" or "healthcare provider*" or "medical provider*" or "healthcare worker*" or "health care professional*" or "professional, health care" or "health personnel").mp. | 327497 |
| 4 | (physician* or doctor* or clinician*).mp. | 1116448 |
| 5 | (nurse* or "nursing staff").mp. | 475378 |
| 6 | exp physicians/ | 180730 |
| 7 | exp nurses/ | 100235 |
| 8 | or/1-7 | 1941378 |
| 9 | (m-health or mhealth or "mobile health" or "health, mobile").mp. | 21417 |
| 10 | (("mobile app*" or "mobile device*" or "medical app*" or "smartphone app*" or "wireless technology" or "mobile technology" or "handheld computer" or "mobile phone") and health).mp. | 28963 |
| 11 | exp mobile applications/ | 13073 |
| 12 | or/9-11 | 47198 |
| 13 | exp "Attitude of Health Personnel"/ or "Attitude of Health Personnel".mp. | 173123 |
| 14 | (adoption OR acceptance OR acceptability OR utilization OR use* OR usage*).mp. | 11557808 |
| 15 | or/13-14 | 11673253 |
| 16 | English.lg. | 33076323 |
| 17 | 8 and 12 and 15 and 16 | 8797 |
| 18 | review.pt | 3181394 |
| 19 | 17 not 18 | 7971 |

Search data: 19 July 2023

Embase

| **Search** | **Query** | **Results** |
| --- | --- | --- |
| #1 | health care personnel'/exp | 1988779 |
| #2 | anesthetists:ab,ti,kw OR dentists:ab,ti,kw OR 'medical staff':ab,ti,kw OR nutritionists:ab,ti,kw OR 'occupational therapists':ab,ti,kw OR pharmacists:ab,ti,kw OR 'physical therapists':ab,ti,kw OR 'advanced practice provider':ab,ti,kw OR 'health educator':ab,ti,kw OR 'mental health care personnel':ab,ti,kw | 145062 |
| #3 | personnel, health':ab,ti,kw OR 'health care provider*':ab,ti,kw OR 'provider, health care':ab,ti,kw OR 'provider, healthcare':ab,ti,kw OR 'healthcare provider*':ab,ti,kw OR 'medical provider*':ab,ti,kw OR 'healthcare worker*':ab,ti,kw OR 'health care professional*':ab,ti,kw OR 'professional, health care':ab,ti,kw OR 'health personnel':ab,ti,kw | 180406 |
| #4 | physician*:ab,ti,kw OR doctor*:ab,ti OR clinician*:ab,ti,kw | 1241757 |
| #5 | nurse*:ab,ti,kw OR 'nursing staff':ab,ti,kw | 413621 |
| #6 | physicians'/exp | 966929 |
| #7 | nurse'/exp | 219104 |
| #8 | #1 OR #2 OR #3 OR #4 OR #5 OR #6 OR #7 | 2991434 |
| #9 | mobile application'/exp | 25083 |
| #10 | m health':ab,ti,kw OR mhealth:ab,ti,kw OR 'mobile health':ab,ti,kw OR 'health, mobile':ab,ti,kw | 14070 |
| #11 | #11 ('mobile app*':ab,ti,kw OR 'mobile device*':ab,ti,kw OR 'medical app*':ab,ti,kw OR 'smartphone app*':ab,ti,kw OR 'wireless technology':ab,ti,kw OR 'mobile technology':ab,ti,kw OR 'handheld computer':ab,ti,kw OR 'mobile phone':ab,ti,kw) AND health:ab,ti,kw | 20210 |
| #12 | #9 or #10 or #11 | 44367 |
| #13 | adoption:ab,ti,kw OR acceptance:ab,ti,kw OR acceptability:ab,ti,kw OR utilization:ab,ti,kw OR use*:ab,ti,kw OR usage*:ab,ti,kw | 11612171 |
| #14 | health personnel attitude'/exp or 'health personnel attitude':ab,ti,kw | 207517 |
| #15 | #13 or #14 | 11758870 |
| #16 | #8 AND #12 AND #15 | 10675 |
| #17 | #8 AND #12 AND #15 AND [english]/lim AND ([article]/lim OR [article in press]/lim OR [conference paper]/lim OR [data papers]/lim OR [short survey]/lim) | 6112 |

Search data: 19 July 2023

**Web of Science**

| **Search** | **Query** | **Results** |
| --- | --- | --- |
| 1 | TS="personnel, health" OR TS="health care provider*" OR TS="provider, health care" OR TS="provider, healthcare" OR TS="healthcare provider*" OR TS="medical provider*" OR TS="healthcare worker*" OR TS="health care professional*" OR TS="professional, health care" OR TS="health personnel" OR TS=Anesthetists OR TS=Dentists OR TS="Medical Staff" OR TS="Pharmacists" OR TS="Physical Therapists" | 212384 |
| 2 | TS=(physician* OR doctor* OR clinician*) | 774314 |
| 3 | TS=nurse* OR TS="nursing staff" | 259725 |
| 4 | #3 OR #2 OR #1 | 1139087 |
| 5 | TS="m-health" OR TS="mhealth" OR TS="mobile health" OR TS="health, mobile" | 18130 |
| 6 | TS="mobile applications" OR TS="mobile app*" OR TS="mobile device*" OR TS="medical app*" OR TS="smartphone app*" OR TS="wireless technology" OR TS="mobile technology" OR TS="handheld computer" OR TS="mobile phone" | 156135 |
| 7 | TS= health | 3141216 |
| 8 | #7 AND #6 | 25340 |
| 9 | #8 OR #5 | 36673 |
| 10 | TS=(adoption OR acceptance OR acceptability OR utilization OR use* OR usage* OR "health personnel attitude") | 17796882 |
| 11 | #10 AND #9 AND #4 | 5720 |
| 12 | #10 AND #9 AND #4 and Article (Document Types) | 4212 |
| 13 | #10 AND #9 AND #4 and Article (Document Types) and English (Languages) | 4126 |

Search data: 19 July 2023

**Search string in Google Scholar**

intitle: (('mhealth*' OR 'm-health*' OR 'mobile health') AND ('personnel, health' OR 'health care provider*' OR 'provider, health care' OR 'provider, healthcare' OR 'healthcare provider*' OR 'medical provider*' OR 'healthcare worker*' OR 'health care professional*' 'professional, health care' OR 'health personnel' OR 'physician*' OR 'doctor*' OR 'clinician*' OR 'nurse*' OR 'nursing staff') AND ('adoption' OR 'acceptance' OR 'acceptability' OR 'utilization' OR 'use*' OR 'usage*' OR 'health personnel attitude'))

Search data: 19 July 2023
