# Supplementary material for: Health Providers’ Perceptions and Experiences of Using mHealth for Chronic Noncommunicable Diseases: Qualitative Systematic Review and Meta-Synthesis
Source: J Med Internet Res. 2023 Sep 12;25:e45437. doi: 10.2196/45437 (PMC10523226; doi:10.2196/45437)
Supplement: Multimedia Appendix 3 [file jmir_v25i1e45437_app3.docx]

Multimedia Appendix 3: Quality assessment of the included studies.

| **Author, Year**^a^ | **➀** | **➁** | **➂** | **➃** | **➄** | **➅** | **➆** | **➇** | **➈** | **➉** |
| --- | --- | --- | --- | --- | --- | --- | --- | --- | --- | --- |
| Korsah et al. 2023 | Y | Y | Y | Y | Y | Y | N | Y | Y | Y |
| Angel et al. 2022 | Y | Y | Y | Y | Y | Y | N | Y | Y | Y |
| Sivakumar et al. 2022 | Y | Y | Y | Y | Y | N | N | Y | Y | Y |
| Giordan et al. 2022 | Y | Y | Y | Y | Y | N | N | Y | Y | Y |
| Melia et al. 2021 | Y | Y | Y | Y | Y | Y | Y | Y | Y | Y |
| Pokhrel et al. 2021 | Y | Y | Y | Y | Y | N | N | Y | Y | Y |
| Dahlhausen et al. 2021 | Y | Y | Y | Y | Y | N | N | Y | Y | Y |
| Silfee et al. 2021 | Y | Y | Y | Y | Y | N | N | Y | Y | Y |
| Patoz et al. 2021 | Y | Y | Y | Y | Y | N | N | Y | Y | Y |
| Furness et al. 2021 | Y | Y | Y | Y | Y | N | N | Y | Y | Y |
| Sarradon-Eck et al. 2021 | Y | Y | Y | Y | Y | N | N | Y | Y | Y |
| Portz et al. 2020 | Y | Y | Y | Y | Y | N | Y | Y | Y | Y |
| Strodl et al.2020 | Y | Y | Y | Y | Y | N | N | Y | U | Y |
| Andrews et al. 2020 | Y | Y | Y | Y | Y | N | N | Y | Y | Y |
| Bally et al. 2020 | Y | Y | Y | Y | Y | Y | N | Y | Y | Y |
| Alwashmi et al.2019 | Y | Y | Y | Y | Y | N | N | Y | Y | Y |
| Han et al. 2019 | Y | Y | Y | Y | Y | N | N | Y | Y | Y |
| Anastasiadou et al. 2019 | Y | Y | Y | Y | Y | N | N | Y | Y | Y |
| Berkowitz et al. 2017 | Y | Y | Y | Y | Y | N | N | Y | N | Y |
| Chiang et al. 2016 | Y | Y | Y | Y | Y | N | N | Y | Y | Y |
| Schneider et al. 2016 | Y | Y | Y | Y | Y | N | N | Y | Y | Y |
| Nundy et al. 2014 | Y | Y | Y | Y | Y | N | N | Y | Y | Y |
| Levine et al. 2014 | Y | Y | Y | Y | Y | N | N | Y | Y | Y |
| Seto et al. 2010 | Y | Y | Y | Y | Y | N | N | Y | Y | Y |

^a^ Reference was shown in the main manuscript.

➀: Is there congruity between the stated philosophical perspective and the research methodology?

➁: Is there congruity between the research methodology and the research question or objectives?

➂: Is there congruity between the research methodology and the methods used to collect data?

➃: Is there congruity between the research methodology and the representation and analysis of data?

➄: Is there congruity between the research methodology and the interpretation of results?

➅: Is there a statement locating the researcher culturally or theoretically?

➆: Is the influence of the researcher on the research, and vice-versa, addressed?

➇: Are participants, and their voices, adequately represented?

➈: Is the research ethical according to current criteria or for recent studies, and is there evidence of ethical approval by an appropriate body?

➉: Do the conclusions drawn in the research report flow from the analysis, or interpretation, of the data?

Y= yes, N= no, U=unclear.
